# Leadership Competencies for Knowledge Translation in Public Health: A consensus study

**DOI:** 10.1093/pubmed/fdab286

**Published:** 2021-07-27

**Authors:** Pablo Rodríguez-Feria, Luis Jorge Hernández Flórez, Katarzyna Czabanowska

**Affiliations:** Department of International Health, CAPHRI - Care and Public Health Research Institute, Maastricht University, The Netherlands; Departamento de Salud Pública, Facultad de Medicina, Universidad de los Andes, Bogotá 11171, Colombia; Departamento de Salud Pública, Facultad de Medicina, Universidad de los Andes, Bogotá 11171, Colombia; Program in Public Health; Schools of Medicine and Government; Universidad de Los Andes; Bogota 11171, Colombia; Department of International Health, CAPHRI - Care and Public Health Research Institute, Maastricht University, The Netherlands; Department of Health Policy and Management, Institute of Public Health, Faculty of Health Sciences, Jagiellonian University, Krakow 31-066, Poland

**Keywords:** competency-based education (MeSH), leadership (MeSH), public health/workforce (MeSH), review literature as topic and Delphi technique (MeSH)

## Abstract

**Background:**

In 2010, 240 billion US dollars was invested worldwide to conduct research for health; unfortunately, 200 billion was misused in the production and reporting of the evidence researched. Universities could facilitate students to acquire leadership competencies to move well-conducted research findings into practical use; this could be an essential move to reduce the misuse of investment.

**Methods:**

A literature review was done based on the Equator Network and Cochrane guidelines, followed by three Delphi rounds to select competencies.

**Results:**

Eleven papers were analysed out of 1121 items and 39/78 identified competencies were prioritized to be presented in the Delphi. Four out of 12 participants accepted to be involved in this project, and 22 competencies reached consensus and stability after three rounds. This framework conceptualizes competencies as the knowledge, skills, attitudes and values. The competencies were framed in four domains: knowledge management, engage diverse others in public health initiatives, training and capacity building/change management and communication.

**Conclusion:**

This framework offers guidance to universities when instructing students with leadership competencies for KT. This project emphasizes that effective leadership should include personal conscience and self-determination values.

## Introduction

It was estimated that in 2010, over 85% of global biomedical research investment was probably wasted due to low priority topics and being dismissive of patients, carers and clinicians; poorly conceived design; non-publication of findings; and biased reporting.^1^^,^^2^

Research for health creates data for organizational culture and patients’ experiences.^3^ These forms of evidence are vital resources for provisioning any health systems, as they can strengthen them by guiding decision-making to improve patient care, workforce quality and health organizations in their structure and behaviours.^4^

Knowledge should lead to action to enhance health systems, but researchers have documented barriers to fulfilling this purpose. Some initial flaws are that the full cycle of biomedical and public health research has not been properly planned, conducted and reported on.^2^ Additionally, knowledge generated by research can be underused, overused or misused when performing evidence-based decision-making.^3^^,^^5–7^

For instance, policy-makers experience barriers such as inadequate infrastructure and systems organizations to transfer knowledge into actions.^7^ They lack knowledge management skills, including accessing research evidence, acquiring skills to appraise evidence and applying research evidence.^5^

As a result, health systems fail to use evidence, which can cause a negative impact in the quantity and quality of life,^5^ financial health resources being limited and ineffective^6^ and increasing healthcare costs.^8^ Moreover, translating research findings into practice may take up to 10 years.^9^

To face these challenges, diverse professionals have created the term ‘knowledge translation’ (KT), which has more than 100 definitions in the health arena.^10^ Specific terms are used in this paper (Table 1).^11^ KT ensures that decision makers at all levels of the health systems, including patients and policy-makers, can use and understand the importance of evidence to inform health-related decision-making. Additionally, the KT strategies vary based on the audience and topic being disseminated.^5^

**Table 1:** Operational definitions for this project

*Concept*	*Definition*	*Reference*
Leadership	‘Leadership is the process of influencing others to understand and agree about what needs to be done and how it can be done effectively, and the process of facilitating individual and collective efforts to accomplish the shared objectives’	Yukl, G. Introduction and overview. In: Yagan, S. (ed). *Leadership in Organizations* [*electronic resource*] (8th edition). Boston, MA; London: Pearson,2013, p 23. ISBN: 978–0–13-277186-3
KT	KT is a process that attempts to reduce the gap between what is known from scientific research and how that knowledge is used by stakeholders with the intention of improving health outcomes and efficiencies of the health care system	^11^
Public health workforce	‘…Full-time professional who provide one or more of the essential public health services, regardless of discipline or work…these individuals hold positions as administrators…’	Begun, J and Peak, S. Why leadership? Why know? In: Peak, S (ed). *Leading Public Health: A Competency Framework* [*electronic resource*] (1^st^ edition). New York: Springer Publishing Company,2014. p 18. eBook ISBN 9780826199072
CBE	‘To summarize, we use “competencies” to refer to broader actions that require multiple skills, knowledge, and particular attitudes or values…’	Begun, J and Peak, S. A framework for public health leadership. In: Peak, S (ed). *Leading Public Health: A Competency Framework* [*electronic resource*] (1^st^ edition). New York: Springer Publishing Company,2014. p 39. Print ISBN 9780826199065 eBook ISBN 9780826199072
Knowledge	‘…key technical and contextual information, theories, and concepts needed to be competent’	Begun, J and Peak, S. A framework for public health leadership. In: Peak, S (ed). *Leading Public Health: A Competency Framework* [*electronic resource*] (1^st^ edition). New York: Springer Publishing Company,2014. p 38. Print ISBN 9780826199065 eBook ISBN 9780826199072
Skills	‘…behavioural practices needed to carry out public health leadership’	Begun, J and Peak, S. A framework for public health leadership. In: Peak, S (ed). *Leading Public Health: A Competency Framework* [*electronic resource*] (1^st^ edition). New York: Springer Publishing Company,2014. p 39. Print ISBN 9780826199065 eBook ISBN 9780826199072
Value	‘…broad preferences concerning appropriate courses of action or outcomes’	Begun, J and Peak, S. Values and traits of public health leaders. In Peak, S (ed). *Leading Public Health: A Competency Framework* [*electronic resource*] (1^st^ edition). New York: Springer Publishing Company,2014. p 53. Print ISBN 9780826199065 eBook ISBN 9780826199072
Attitudes	‘a learned, global evaluation of an object(person, place or issue) that influences thought and action’	Perloff, R. Attitudes: definition and structure. In: Bathgate, L (ed). *The Dynamics of Persuasion: Communication and Attitudes in the 21st century [electronic resource]* (2^nd^ edition)*.* London: Lawrence Erlbaum Associates, Publishers Mahwah,2003. p 39. ISBN 0-8058-4087-7 (case alk. paper)—ISBN 0-8058-4088-5 (pbk alk. paper)

Strifler *et al*. studied KT for preventing and/or management of chronic diseases. To improve healthcare practice and policies, they conducted a scoping review and identified 596 studies reporting 159 KT theories, models or frameworks from 2000 to 2016. Interventions could be considered for more than one stakeholder (e.g. financial/regulatory entities).^12^

Developing KT frameworks is not only enhancing the importance of the discipline itself but it also contributes to supporting public health workforces with competencies to reduce the ‘know - do gap’,^13–15^ and Mallidou *et al*. and Bayley *et al*. mentioned that leadership was a key skill for conducting KT.^14^^,^^15^

A Public health workforce with ‘leadership competencies’ can contribute to promoting KT by constructing meaning, establishing frameworks to understand and apply new knowledge and by creating and sharing a new vision with others.^16^

It is paramount to develop a public health workforce with the necessary leadership competencies to enhance KT, and universities have a central role in teaching and training students. Frameworks should be consistent with the World Health Organisation’s (WHO) public health workers’ functions/operations^17^ and prioritize the most relevant competencies for the foundations.

However, these frameworks for instructing the public health workforce based on competency-based education (CBE) for leadership lack competencies needed for KT; additionally, the previous KT frameworks display leadership as a skill. Therefore, the goal is to develop and propose public health leadership competencies for KT using a literature review and consensus study. This project defines competencies as the values, skills, attitudes and knowledge.

## Methods

### Literature review

The literature review used a systematic approach based on the Equator Network for systematic review (PRISMA) and Cochrane Handbook for systematic review version 5.2.0. This review gathered published and unpublished literature in English and Spanish, and the Supplementary File 1 represented the search strategy. The last search was done on 21 March 2019.

Literature was excluded by phases (duplicated literature, title, abstract and exclusion and inclusion criteria) (Table 2) and by two authors PARF and LJHF, and when disagreements were present, PARF and KC resolved them.

**Table 2:** Inclusion and exclusion criteria for the literature (first step) and selecting participants for the Delphi technique (second part)

*Inclusion criteria*, *literature review*	*Exclusion criteria*, *literature review*
Study design: Literature that is published or unpublished. Observational studies, qualitative research, systematic reviews, randomized trials, expert opinions, policy documents, abstracts, conference papers that use quantitative and qualitative	No exclusion criteria for study design
Topic: Studies that assessed competencies for leadership in public health workforce or studies that evaluated (knowledge, skills, values or attitudes). Additionally, studies that link competencies for public health leaders and knowledge translation	Topic: Studies that do not evaluate competencies or its components, competencies that are not related with public health and competencies that are not focused on leadership in this workforce
Limit: The studies have been written in Spanish or English and being published in or after the year 2005 when competency models about leadership in public health start to be documented in the literature	Limit: Evidence published or unpublished that one cannot get full access to or is not published in English or Spanish. Some databases the publication date cannot be embedded in the searching strategy, thereby studies published before 2005 will be removed
Quality assessment – Quantitative: rate ‘strong’ or ‘moderate’ – Qualitative: rate ¨yes¨ or ‘I cannot tell’ – Grey literature: ‘yes’ or ‘?’ – Mix methods: rate ‘yes’ or ‘I cannot tell’	Quality assessment – Quantitative: rate sections with ‘weak’ – Qualitative: rate ‘no’ – Grey literature: ‘no’ – Mix methods: rate ‘no’
*Inclusion criteria, Delphi technique*	*Exclusion criteria, Delphi technique*
1. The participant has been recognized by having publication from this research database or being suggested by the researcher 2. The participant is working on leadership competencies for public health workforce, or the participant has knowledge and/or experience about knowledge translation based on his or her professional webpage 3. Willingness and capacity to participate	1. The participant did not reply to the emails 2. The person rejected invitation to participate in the project 3. The participant quit involvement (any round)

Assessing each paper’s quality was conducted by PARF and revised by LJHF, using the following documents: the Effective Public Health Practice Project toolkits, which include the Quality Assessment Tool for Quantitative Studies^18^; the Critical Appraisal Skills Programme Qualitative Research Checklist^19^; the Authority, Accuracy, Coverage, Objectivity, Date, Significance checklist for grey literature^20^ and the Mixed Methods Appraisal Tool Version 2011.^21^

Papers that accomplished the criteria were read to extract their competencies, which were clustered in domains, and they were re-phrased, combined and eliminated based on previous KT frameworks that were identified in the review. Lastly, the core competencies were prioritized based on their role in conducting KT.

Additionally, the researchers considered how each competency fostered the WHO’s Essential Public Health Functions or Operations frameworks in diverse regions, including Latin America and the Caribbean, Eastern Mediterranean Region and the European Region.^22^ This step was done by PARF and LJHF, individually, and KC revised both lists to prioritize, merge and rephrase competencies for the Delphi.

### Delphi technique

Convenience and snowballing sampling techniques were used to invite participants. The process started when PARF revised the papers from the review and had extracted their authors’ affiliations. Afterwards, each author’s official institution website was scanned to (i) obtain authors’ interests and professional profile, (ii) acquire authors’ email contact and (iii) trace their co-workers to determine their working experience.

Lastly, a list of participants was created and provided to KC and LJHF to re-examine the participants and to include individuals who they might know that work on these arenas. Participants were selected based on a criterion of experience in leadership and/or KT in the public health workforce. An email was sent to them, which explained the project’s purpose and invited them and their contacts to be involved.

This Delphi study used a four-point Likert-chart scale,^23^ one ‘strongly *disagree*’, two ‘*disagree*’, three ‘*agree*’ and four ‘strongly agree’ for ranking the competencies. This scale did not include middle point, which could be interpreted as ‘neutral’ or ‘undecided’. In addition, the fifth option was ‘no comment opinion’ if participants felt they could not rank a statement in the questionnaire. If a participant did not mark a competency, the researcher considered it as a no comment opinion.

Consensus was defined to at least 70% rate three or higher on a four-point Likert-type scale, the median being ≥3.25 and the interquartile range (IQR) ≤ 1.75 in two successive iterations.^22^^,^^24^ The researchers used Survey Monkey for this phase.

Participants were encouraged to provide their arguments to keep, change, add or remove competencies and their domains throughout each round. They received documentation that contained the percentage of the competencies that were marked agree (three) and strongly agree (four), the median (central tendency), the IQR (dispersion to aggregate data), competencies graphical representation (boxplot) and participants’ comments. Competencies were grouped by domains, and the participants received the project’s protocol and the manuscript before submission for publication.

Ethical approval was granted by the University of Sheffield (Supplementary Material 2).

## Results

**Fig. 1:**
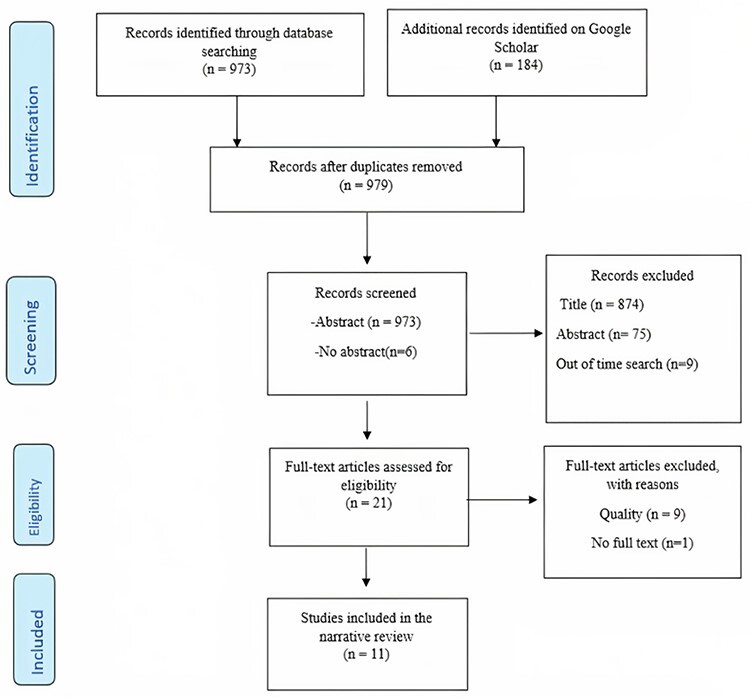
Equator Network: PRISMA flow diagram for the identification, screening, eligibility and included papers that were identified in the search strategy.

The researchers identified 1121 papers, and after applying the study’s criteria, 11 documents were analysed in the literature review (see Fig. 1 and Supplementary File 3 for a description of each)*.* Most authors utilized triangulation methods to collect data (*n* = 6), for instance literature review, consensus development panel and Delphi survey. Seventy-eight competencies were identified, and 39 competencies were prioritized to be used in the Delphi section.

Twelve potential individuals were emailed to participate in the second segment, and four participants were willing to be part of it. The response rate was 100% for the first iteration and 75% for the other two rounds. For the first round, seven domains were adapted from Czabanowska *et al*.’s paper, which was part of the literature review.^17^ These were (i) political leadership, (ii) knowledge translation and mobilization, (iii) research and evidence, (iv) communication and impact, (v) collaboration and engagement, (vi) system thinking and change and (vii) professional ethical practice.

In the first round, the participants were advised to give their opinion on the 39 competencies within the seven domains. However, the participants did not make a comment about the relationship between competencies and domains, meaning that the authors did not know their perspectives towards them. The participants suggested three more competencies (39 + 3 = 42 competencies).

For the second round, 42 competencies were provided to the participants based on their comments; they had to rate them and comment on which domain they would suit. The participants were able to suggest the domains from the first round or provide a new one. Participants mentioned the domain ‘management role’, which included three competencies, and the domain ‘contextual analysis of readiness for KT’ with one competency, but 38 competencies did not have a domain.

The third round followed the same procedure as the second one, and at the end, 22 competencies reached consensus and stability (Table 3). Participants proposed two domains for a limited number of competencies, and these were inadequate to establish a framework.

Consequently, according to the literature review, four domains were selected: (i) knowledge management; (ii) engage diverse others in public health initiatives; (iii) training and capacity building/change management and (iv) communication (Supplementary Material 4). Each competency was paired within a domain by PARF and had been presented to the others to agree the best fit for each one. The disagreements were resolved by discussion.

Competencies were framed to combine knowledge, attitudes, skills and values. For instance, competency 12 highlights values’ importance for leadership in the public health workforce: ‘Model effective leadership values including personal conscience Values and self determination Values’.

## Discussion

### Main finding of this study

Literature has documented the importance of leadership and KT for the public health workforce, as leaders are active agents who improve health of individuals by recognizing, highlighting and connecting relevant stakeholders to confront health challenges.

This project proposed a framework with 22 competencies in four domains: (i) knowledge management (**D1**) (ii) engaging diverse others in public health initiatives (**D2**), (iii) training and capacity building/change management **(D3)** and (iv) communication (**D4**). This research used a literature review to select competencies that were aligned with the WHO public health functions,^22^ followed by a Delphi study.

### What is already known on this topic

This framework compares to previous literature about KT in public health workforce such as Straus *et al*., 2011, Mallidou *et al*., 2018 and Bayley *et al*., 2018. The main similarity is that each framework is focused on competencies, which are the expected outcomes when teaching and training individuals. Additionally, D1’s and D2’s competencies are consistent with at least one of the earlier frameworks.

For instance, ‘Have a clear understanding of the governance and stewardship of the involved entities and the knowledge translation initiatives’ was recognized as a core competency by former frameworks, and it was included in two systematic reviews (Table 3).^25^^,^^26^

**Table 3:** Leadership Competencies Framework for Knowledge Translation in the Public Health Workforce (domains and their competencies)

*Number*	*Domain*	*Competency*
1	Knowledge management	*Have* a clear understanding of the governance and stewardship of the involved entities and the knowledge translation initiatives
2		Have the skills to diagnose the existing professional and organizational context when implementing knowledge mobilization projects
3		Define appropriate process and outcome measures to assess translational efforts
4		Show understanding of and skilful use of research, specifically operational and implementation research in complex environments
5		Show analytical skills, including policy analysis and development skills, stakeholder analysis, critical thinking and strategic use of data
6	Engage diverse others in public health initiatives	*Identify* and engage stakeholders in interdisciplinary, transdisciplinary or intersectoral projects to improve public health
7		Track the dynamic social processes and networks through patient and public involvement (e.g. Organized Civil Society) to make contributions to health improvements
8		Respect diverse cultures and build upon the strength of diversity to bring about innovation and added value in the work environment
9		share views in a non-judgemental, non-threating way
10		Effectively use negotiation skills to mediate disputes, find appropriate and workable solutions while creating new opportunities for partnership and collaboration
11		Foster patient and public involvement across knowledge translation process
12	Training and capacity building/change management	*Model* effective leadership values including personal conscience values (e.g. fairness, trustworthiness, conscientiousness, patience or truth and honesty) Additionally, self-determination Values (e.g. purpose, motivation, power or resilience)
13		Offer opportunities for collaborative learning and quality improvement
14		Determine different roles in a KT team and develop, match or strengthen workers’ competencies to accomplish task requirements
15		Advocate for learning opportunities within the organization
16		Serve as a driving force for change, including strategies of change and manage staff to effectively deal with change
17		Develop agendas using participatory approaches by giving voice to relevant stakeholders and making sure that these agendas are reviewed and updated periodically
18	Communication	*Translate* broad strategies into practical terms for others
19		Demonstrate effective written and oral communication and presentation skills to support knowledge translation in organizations
20		Depending on your role provide an environment conductive to opinion sharing with diverse cultures, disciplines and professions
21		Synthesize and integrate divergent views to conduct into the knowledge translational process in other to achieve shared organizational goals
22		Effectively share information and responsibility at different organizational levels in pursuit of population-based goals

Orton *et al*. analysed 18 studies to explore the use of research in public health decision-making process, and the authors concluded that researchers should align evidence with the current and future policy environment.^25^ Likewise, Innvaer *et al*. studied 24 papers to determine health policy-makers’ perception of their use of evidence. Results showed that the documents that included a summary with precise recommendations, personal contact between researchers and policy-makers, and good quality research that aligned with policies or self-interest facilitated policy-makers’ use of evidence.^26^

Differences across earlier frameworks were as follows: (i) definitions of competencies and (ii) the content of the methods sections. Competency’s definition in former frameworks was referred to as skills^15^ or as knowledge, attitudes and skills without values.^14^ One framework did not define competency, but it was contextualized as ‘knowledge and understanding’ or ‘capacity to/in’.^13^

Secondly, the methods may well have differed, as Straus *et al*. did not provide a methods section^13^; thereby, it was not possible to judge how the literature search was conducted and how the two frameworks were selected. Bayley *et al*. used a three-stage process: identification, synthesis and production of the final competency set^15^. The first step was done by a consulting committee member from the UK and Canada, which took place at the knowledge mobilization forum in 2015. Mallidou *et al*. employed a scoping review that included published and unpublished literature that resulted in 21 published articles and 52 grey literature items.^14^

Using domains to organize competencies was another difference. Strauss *et al*. did not use domains in their framework^13^; one possible reason was that they used four competencies in their training initiative. Malliduo *et al*. on the other hand distributed competencies into four domains: knowledge, skills, attitudes and other.^14^

Lastly, Bayley *et al*. had 11 domains, including communication, which was used in this project, and another 2 domains were merged into one domain in this paper (change management and training capacities).^15^

There was a divergence in domains between Bayley *et al*.^15^ and this project. The difference was in how the interaction with others was understood, as this project mentioned the domain ‘Engage diverse others in public health work’ as ‘searching broadly for partners, understanding their world view and practical circumstances, outlining potential responses, and identifying the right partners for a particular activity’.

In contrast, Bayley *et al*.’s ‘Managing partnership/relationship’ domain was ‘maintaining partnership and sustaining relationships with engaged external/internal stakeholders’.^15^^, p.731^ This research focused on the process to engage others: (i) search and identify others and (ii) understand and interact with them, and Bayley’s project was concentrated on outcomes such as maintain, sustain or engage partnerships or relationships.

### What this study adds

This project adds new insight about CBE for leadership and KT in the public health workforce. First, it defines and contextualizes competencies as the combination of knowledge, skills, attitudes and values. Secondly, while values are important and they are represented in competency 12, they have been poorly analysed in earlier frameworks.^13–15^ Malliduo *et al*. only mentioned ‘having trust about the character or integrity about others’.^14^

To provide some light, core leadership values can be divided into personal conscience and self-determination.^27^ The former is related to who I am, and the latter reflects what I am going to do based on who I am. Personal conscience values include: fairness, trustworthiness, patience, truth and honesty, excellence, integrity and forgiveness, among others. Self-determination includes purpose, motivation, energy, courage and resilience, among others. For instance, Innvaer’s systematic review concluded that mistrust between policy-makers and research was a common barrier for using research evidence to inform policy-making decisions.^26^

Similarly, value-based leadership improved leaders’ effectiveness and therefore success in organizations when performing KT. One explanation is that leaders communicated their values to employees, and this might foster a strong work ethic, cooperative behaviour and commitment to pursue organizations’ goals. For example, if leaders’ values overlapped with organizations’ values, leaders’ actions would be viewed by workers as the standard conduct for achieving workers and organizational targets.^28^^,^^29^

### Limitations of this study

This study has limitations. The search strategy did not use each KT synonym that has been documented in the literature. However, the Delphi technique should ameliorate this limitation by relying on participants who worked and had interest in KT and/or leadership.

The information provided by the selected articles varied in quality concerning the content of methods section. Based on the quality toolkits, we labelled this information as ‘I cannot tell’ when authors partially reported their research (Table 2) and excluded it when the reports did not contain any information. We did not contact authors to clarify papers’ information.

The Delphi participants were selected based on the hits from the literature review and professional networking; therefore, it was possible that some potential participants were not invited to participate in the project. Four out of 12 participants participated and only 3 of them completed all rounds, which is a limitation. It is important to highlight that any competency framework could change according to consensus definition and participant numbers. Consequently, this study provided the competencies that were included in the framework and the competencies that were excluded, and this framework offers a guidance to universities that want to change/upgrade their curricula.

Supplementary File 5 shows competencies that did not reach consensus in our study but might be relevant for others. Competencies 2, 14, 15 and 17 demonstrate that the public health workforce should be able to advocate for their initiatives, influence policies, and resource allocation at the local, national and/or international levels by (i) understanding the nature of public health issues, (ii) making decisions based on values, priorities and resources, (iii) considering political, economic and social systems and (iv) contemplating the policy options to engage in systematic change. These competencies have been highlighted in KT and leadership arenas for this workforce.^30^^,^^31^^,^^32^ For instance, the Canadian government determines core competencies such as analysing ethical, political, scientific, socio-cultural and economic contexts, and developing key values in planning and implementing public health programs and policies.^30^

#### Recommendations

This framework can be used by universities as a guidance for CBE in the public health workforce across the education continuum. Therefore, the authors provide some recommendations based on their experience in this study.

1. CBE should encompass the knowledge, skills, attitudes and values when creating university curricula at bachelor, master and PhD level in public health professions.
2. CBE should integrate leadership and KT in order to face the challenge of improper use of resources when conducting, implementing, sustaining and evaluating research for health.
3. It seems that it is necessary to conduct rigorous studies on leadership and KT competencies and evaluate how these competencies contribute to developing effective public health leaders able to conduct KT activities.

## Conclusions

The public health workforce needs to have leadership competency framework to stimulate the KT process, and it should be included in the CBE. Competency composition varies across publications, and none of them includes values in their frameworks. Values have two categories, personal conscience and self determination, which contribute to strengthening leadership and KT process across actors. Therefore, it seems necessary to not only prioritize technical–scientific competencies but also the ones that are based on value principles.

## Supplementary Material

Supplementary_fdab286

## Declarations

Informed consent was obtained from all individual participants included in the study by Charlotte Claxon on behalf of the School of Health and Related Research Ethics Committee Administrator at the University of Sheffield. They received an email that explained that this manuscript would be published in a journal and they could read and provide feedback about it.

## Notes

**Pablo Andres Rodriguez,** PhD Candidate

**Luis Jorge Hernandez Florez,** Associate Professor

**Katarzyna Czabanowska,** Associate Professor
