# Supplementary material for: Leadership Competencies for Knowledge Translation in Public Health: A consensus study
Source: J Public Health (Oxf). 2021 Jul 27;44(4):926–35. doi: 10.1093/pubmed/fdab286 (PMC9715294; doi:10.1093/pubmed/fdab286)
Supplement: Supplementary_fdab286 [file supplementary_fdab286.docx]

**Supplementary file 1 Search strategy for each database**

The search strategy for each database, the hits and the date when the search was performed is displayed in this supplementary material. The search strategy is based on controlled vocabulary such as MeSH, EMTREE or ERIC Descriptors, key words and their synonyms and syntax searching (e.g., truncation ¨*¨) and boolean operators.

OpenSIGLE does not have controlled vocabulary or range of year of publication, but it offers operators (and, or, and not) and syntax (e.g., quotation marks). Google scholar offers advance search, which includes some domains such as exact phrase, at least one of the words, title and abstract. However, it does not contain controlled vocabulary or syntax such as truncation.

1. *Medline via (PubMed)*

| ***Date.*** | ***Search Strategy.*** | ***Hits.*** |
| --- | --- | --- |
| ^13st^/March/2019 | 1. Leadership [Mesh] 2. [Students, Public Health](https://www.ncbi.nlm.nih.gov/mesh/68040443)[Mesh] 3. [Education, Public Health Professional](https://www.ncbi.nlm.nih.gov/mesh/68040381) [Mesh] 4. Health Professions Education[ti,ab] 5. Public health leader* [ ti,ab] 6. #1 OR # 2 OR # 3 OR # 4 OR #5 7. Leadership Competenc* [ti,ab] 8. public health leadership competenc*[ti,ab] 9. Competency-based education [ti,ab] 10. Knowledge [ti,ab] 11. Skill* [ti,ab] 12. Attitude* [ti,ab] 13. Value* [ti,ab] 14. Trait*[ ti,ab] 15. # 7 OR # 8 OR # 9 OR # 10 OR # 11 OR # 12 OR # 13 OR # 14 16. Language Spanish 17. Language English 18. #16 OR #17 19. # 6 AND #15 AND # 18 AND Date publication 1/1/2005 to present | 4770 |
| 15^th^/March/2019 | 1. Leadership [Mesh] 2. [Students, Public Health](https://www.ncbi.nlm.nih.gov/mesh/68040443)[Mesh] 3. [Education, Public Health Professional](https://www.ncbi.nlm.nih.gov/mesh/68040381) [Mesh] 4. Health Professions Education[ti,ab] 5. Public health leader* [ ti,ab] 6. #1 OR # 2 OR # 3 OR # 4 OR #5 7. Leadership Competenc* [ti,ab] 8. public health leadership competenc*[ti,ab] 9. Competency-based education [ti,ab] 10. # 7 OR #8 OR #9 11. Language Spanish 12. Language English 13. #11 OR #12 14. # 6 AND # 10 AND #13 AND Date publication 1/1/2005 to present | 225 |
| 15^th^/March/2019  ^( Included in the database)^ | 1. Leadership [Mesh] 2. [Students, Public Health](https://www.ncbi.nlm.nih.gov/mesh/68040443)[Mesh] 3. [Education, Public Health Professional](https://www.ncbi.nlm.nih.gov/mesh/68040381) [Mesh] 4. Health Professions Education[ti,ab] 5. Public health leader* [ ti,ab] 6. #1 OR # 2 OR # 3 OR # 4 OR #5 7. Leadership Competenc* [ti,ab] 8. public health leadership competenc*[ti,ab] 9. Competency-based education [mesh] 10. Knowledge trans*[ti,ab] 11. Knowledge broker* [ti,ab] 12. # 7 OR # 8 OR #9 OR #10 OR#11 13. Language Spanish 14. Language English 15. #13 OR #14 16. #6 AND #12 AND #15 AND Date publication 1/1/2005 to present | 421 |
|  |  |  |

1. *Embase via (OVID)*

| Date | Search strategy | Hits |
| --- | --- | --- |
| 14^th^ /March/2019 | 1. Leadership[subject heading ] 2. [public health student](https://ovidsp-tx-ovid-com.ezproxy.ub.unimaas.nl/sp-3.32.2a/ovidweb.cgi?&Controlled+Vocabulary=Mapping%7c0&Return=mapping&S=HIIFFPHDAODDPHMFNCDKMGJCBNFBAA00)[ subject heading] 3. "‘Public health leader*¨.ab, ti 4. 1 or 2 or 3 5. Curriculum [Subject heading] 6. "‘[competenc* based education](https://ovidsp-tx-ovid-com.ezproxy.ub.unimaas.nl/sp-3.32.2a/ovidweb.cgi?S=HIIFFPHDAODDPHMFNCDKMGJCBNFBAA00&Controlled+Vocabulary=thes+competency-based+education&)’".ti,ab. 7. [school health education](https://ovidsp-tx-ovid-com.ezproxy.ub.unimaas.nl/sp-3.32.2a/ovidweb.cgi?&Controlled+Vocabulary=Mapping%7c40&Return=mapping&S=HIIFFPHDAODDPHMFNCDKMGJCBNFBAA00) [ Subject heading] 8. Knowledge. ti,ab. 9. Skill.ti,ab. 10. Attitude.ti,ab. 11. Value.ti,ab. 12. Trait.ti,ab. 13. "‘Knowledge trans*’".ti,ab. 14. "‘Knowledge broker*’".ti,ab. 15. 5 or 6 or 7 or 8 or 9 or 10 or 11 or 12 or 13 or 14 16. Spanish lg 17. English lg 18. 16 or 17 19. 4 and 15 and 19 and Publication year 2005 to current | 7788 |
| 15^th^ / 03/2019 | 1. Leadership.sh. 2. [public health student](https://ovidsp-tx-ovid-com.ezproxy.ub.unimaas.nl/sp-3.32.2a/ovidweb.cgi?&Controlled+Vocabulary=Mapping%7c0&Return=mapping&S=HIIFFPHDAODDPHMFNCDKMGJCBNFBAA00).sh. 3. "‘Public health leader**’".ti, ab. 4. 1 or 2 or 3 5. Curriculum.sh. 6. "‘[competenc* based education](https://ovidsp-tx-ovid-com.ezproxy.ub.unimaas.nl/sp-3.32.2a/ovidweb.cgi?S=HIIFFPHDAODDPHMFNCDKMGJCBNFBAA00&Controlled+Vocabulary=thes+competency-based+education&)*’".ti,ab. 7. [school health education](https://ovidsp-tx-ovid-com.ezproxy.ub.unimaas.nl/sp-3.32.2a/ovidweb.cgi?&Controlled+Vocabulary=Mapping%7c40&Return=mapping&S=HIIFFPHDAODDPHMFNCDKMGJCBNFBAA00).sh. 8. "‘Knowledge trans*’".ti,ab. 9. "‘Knowledge broker*’".ti,ab. 10. 5 or 6 or 7 or 8 11. Spanish.lg. 12. English .lg. 13. 11 or 12 14. 4 and 10 and 13 and Publication year 2005 to current | 2203 |
| 18^th^ /03/2019  ^( Included in the database)^ | 1. Leadership.sh. 2. [public health student](https://ovidsp-tx-ovid-com.ezproxy.ub.unimaas.nl/sp-3.32.2a/ovidweb.cgi?&Controlled+Vocabulary=Mapping%7c0&Return=mapping&S=HIIFFPHDAODDPHMFNCDKMGJCBNFBAA00).sh. 3. Public health leader*. ti, ab. 4. 1 or 2 or 3 5. Competency-based education.kw. 6. "‘[Competenc* based education](https://ovidsp-tx-ovid-com.ezproxy.ub.unimaas.nl/sp-3.32.2a/ovidweb.cgi?S=HIIFFPHDAODDPHMFNCDKMGJCBNFBAA00&Controlled+Vocabulary=thes+competency-based+education&)".ti,ab. 7. [School health education](https://ovidsp-tx-ovid-com.ezproxy.ub.unimaas.nl/sp-3.32.2a/ovidweb.cgi?&Controlled+Vocabulary=Mapping%7c40&Return=mapping&S=HIIFFPHDAODDPHMFNCDKMGJCBNFBAA00) .sh. 8. "‘Knowledge trans*’".ti,ab. 9. "‘Knowledge broker*’"ti,ab. 10. 5 or 6 or 7 or 8 or 9 11. Spanish lg 12. English lg 13. 11 or 12 14. 4 and 10 and 13 and Publication year 2005 to current | 226 |

*C.Education Resources Information Center( ERIC) via EBSCO HOST*

| Date | Search strategy | Hits |
| --- | --- | --- |
| 14^th^ /03/2019 | 1. Leadership DE 2. Leadership training DE 3. Student leadership DE 4. Public Health DE 5. ¨[public health student](https://ovidsp-tx-ovid-com.ezproxy.ub.unimaas.nl/sp-3.32.2a/ovidweb.cgi?&Controlled+Vocabulary=Mapping%7c0&Return=mapping&S=HIIFFPHDAODDPHMFNCDKMGJCBNFBAA00)¨ 6. ¨Public health leader¨ 7. 1 or 2 or 3 or 4 or 5 or 6 8. Curriculum DE 9. ¨[competency-based education](https://ovidsp-tx-ovid-com.ezproxy.ub.unimaas.nl/sp-3.32.2a/ovidweb.cgi?S=HIIFFPHDAODDPHMFNCDKMGJCBNFBAA00&Controlled+Vocabulary=thes+competency-based+education&)¨ AB TI 10. ¨ [Public health education](https://ovidsp-tx-ovid-com.ezproxy.ub.unimaas.nl/sp-3.32.2a/ovidweb.cgi?&Controlled+Vocabulary=Mapping%7c40&Return=mapping&S=HIIFFPHDAODDPHMFNCDKMGJCBNFBAA00)¨ AB TI 11. Ability DE 12. Knowledge AB TI 13. Attitudes DE 14. Values 15. Trait 16. ¨Knowledge translation or¨ Knowledge transfer ¨ or ¨Knowledge broker” AB TI 17. 8 or 9 or 10 or 11 or 12 or 13 or 14 or 15 or 16 18. 7 or 17 19. Spanish or English [language] 20. Publication year 2000 to 2018 | 601 |
| 15^th^ /03/2019  ^( Included in the database)^ | 1. Leadership DE 2. Leadership training DE 3. Student leadership DE 4. Public Health DE 5. ¨[public health student](https://ovidsp-tx-ovid-com.ezproxy.ub.unimaas.nl/sp-3.32.2a/ovidweb.cgi?&Controlled+Vocabulary=Mapping%7c0&Return=mapping&S=HIIFFPHDAODDPHMFNCDKMGJCBNFBAA00)¨ AB TI 6. ¨Public health leader¨ AB TI 7. 1 or 2 or 3 or 4 or 5 or 6 8. Curriculum DE 9. ¨Competence-Based Education¨ or ¨[competency-based education](https://ovidsp-tx-ovid-com.ezproxy.ub.unimaas.nl/sp-3.32.2a/ovidweb.cgi?S=HIIFFPHDAODDPHMFNCDKMGJCBNFBAA00&Controlled+Vocabulary=thes+competency-based+education&)¨ TI AB 10. ¨ [Public health education](https://ovidsp-tx-ovid-com.ezproxy.ub.unimaas.nl/sp-3.32.2a/ovidweb.cgi?&Controlled+Vocabulary=Mapping%7c40&Return=mapping&S=HIIFFPHDAODDPHMFNCDKMGJCBNFBAA00)¨ TI AB 11. ¨Knowledge translation or¨ Knowledge transfer ¨ or ¨Knowledge broker” TI AB 12. Ability DE 13. 8 or 9 or 10 or 11 or 12 14. Spanish or English [language] 15. Publication year 2005 to 2018 | 184 |

*D.OpenGREY*

| Date | Search strategy^^[[1]](#footnote-1)^^ | Hits |
| --- | --- | --- |
| 14^th^ /3/2019  ^( Included in the database)^ | 1. Leadership 2. ¨public health student*¨ 3. ¨public health leader*¨ 4. 1 OR 2 OR 3 5. ¨Competenc*-based education¨ 6. ¨ competence*¨ or ¨ability¨ 7. ¨Knowledge Trans*¨ or ¨ knowledge broker*¨ 8. 5 OR 6 OR 7 9. Language English^^[[2]](#footnote-2)^^ | 106 |

*E. Google scholar*

| Date | Search Strategy | Hits |
| --- | --- | --- |
| 18^th^/03/2019  ^( Included in the database)^ | 1. with the exact phrase: competency-based education 2. with at least one of the words: public health 3. where my words occur: in the title of the article 4. Return articles dated between: 2005-2019 5. [Search English and Spanish pages](https://scholar.google.com/scholar?lr=lang_en\|lang_es&q=allintitle:+public+OR+health+%22competency+based+education%22&hl=en&as_sdt=0,5&as_ylo=2005&as_yhi=2019) 6. Include patents, include citations. | 53 |
| 18^th^/03/2019 | 1. with all of the words: knowledge translation 2. with the exact phrase: competency-based education 3. where my words occur: in the title of the article 4. Return articles dated between 2005-2019 5. [Search English and Spanish pages](https://scholar.google.com/scholar?lr=lang_en\|lang_es&q=allintitle:+public+OR+health+%22competency+based+education%22&hl=en&as_sdt=0,5&as_ylo=2005&as_yhi=2019) 6. Include patents, include citations. | 0 |
|  | 1. with all of the words: knowledge transfer 2. with the exact phrase: competency-based education 3. where my words occur: in the title of the article 4. Return articles dated between 2005-2019 5. [Search English and Spanish pages](https://scholar.google.com/scholar?lr=lang_en\|lang_es&q=allintitle:+public+OR+health+%22competency+based+education%22&hl=en&as_sdt=0,5&as_ylo=2005&as_yhi=2019) 6. Include patents, include citations. | 0 |
| 18^th^/03/2019  ^( Included in the database)^ | 1. with all of the words: curriculum 2. with the exact phrase knowledge translation 3. where my words occur: in the title of the article 4. Return articles dated between 2005-2019 5. [Search English and Spanish pages](https://scholar.google.com/scholar?lr=lang_en\|lang_es&q=allintitle:+public+OR+health+%22competency+based+education%22&hl=en&as_sdt=0,5&as_ylo=2005&as_yhi=2019) 6. Include patents, include citations. | 9 |
| 18^th^/03/2019  ^( Included in the database)^ | 1. with the exact phrase: competency-based education 2. with at least one of the words: knowledge OR transfer OR translation OR broker OR student OR health 3. where my words occur: in the title of the article 4. Return articles dated between 2005-2019 5. [Search English and Spanish pages](https://scholar.google.com/scholar?lr=lang_en\|lang_es&q=allintitle:+public+OR+health+%22competency+based+education%22&hl=en&as_sdt=0,5&as_ylo=2005&as_yhi=2019) 6. Include patents, include citations. | 89 |
| 21^st^/03/2019 | 1. with all of the words: Public Health 2. with the exact phrase: competency framework 3. where my words occur: in the title of the article 4. Return articles dated between 2005-2019 5. [Search English and Spanish pages](https://scholar.google.com/scholar?lr=lang_en\|lang_es&q=allintitle:+public+OR+health+%22competency+based+education%22&hl=en&as_sdt=0,5&as_ylo=2005&as_yhi=2019) 6. Include patents, include citations. | 13 |
| 21^st^/03/2019  18^th^/03/2019 | 1. with all of the words: Public Health 2. with the exact phrase: competency model 3. where my words occur: in the title of the article 4. Return articles dated between 2005-2019 5. [Search English and Spanish pages](https://scholar.google.com/scholar?lr=lang_en\|lang_es&q=allintitle:+public+OR+health+%22competency+based+education%22&hl=en&as_sdt=0,5&as_ylo=2005&as_yhi=2019) 6. Include patents, include citations. | 20 |

**Supplementary file 2. Ethical approval**


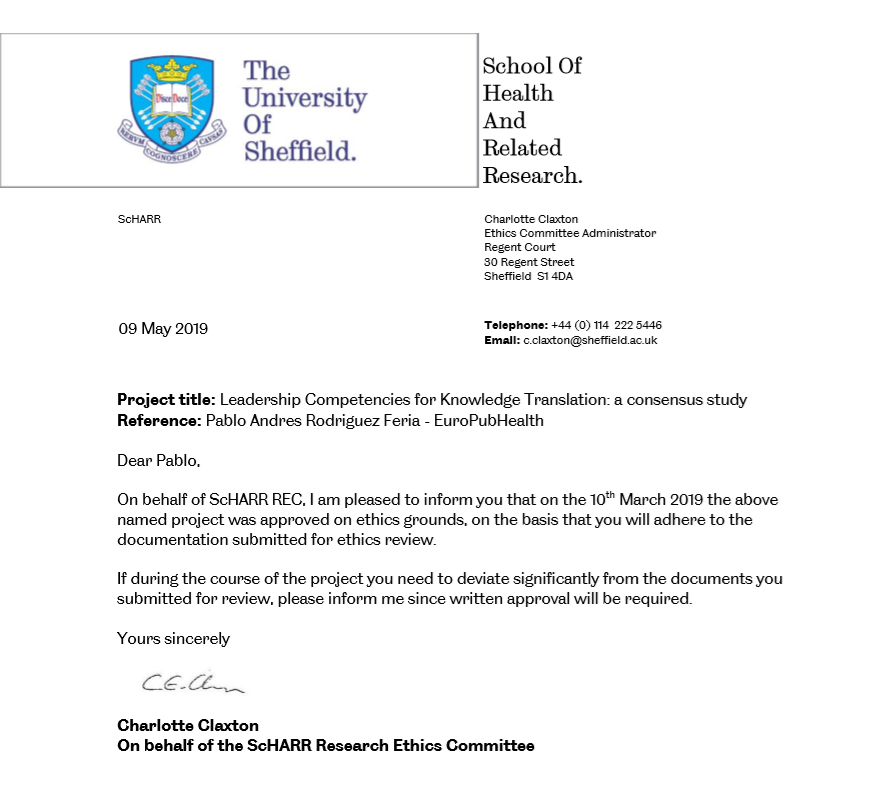


**Supplementary file 3. Characteristics of Included Studies**

Table of data describing characteristics of included studies.

[ order by date of publication from newest to oldest]

1. Learning from the emergence of NIHR Collaborations for Leadership in Applied Health Research and Care (CLAHRCs): a systematic review of evaluations. August 15^th^ ,2018.

| Contact detail | Paul M Wilson: paul.wilson@manchester.ac.uk |
| --- | --- |
| Author information | University of Manchester, Manchester, UK NIHR Collaboration for Leadership in Applied Health Research, and Care Greater Manchester, Manchester, UK. |
| Objective | The aim of this review is to synthesise learning from evaluations of the Collaborations for Leadership in Applied Health Research and Care. |
| Methods | Systematic review (PROSPERO Registration number CRD42016042945); we have focused on published papers, and All the searches were restricted to English language only and were conducted in June 2016; update searches were conducted up to June 2018 using the same search terms and databases. Inclusion criteria: Any published empirical papers drawing on data from an evaluation of CLAHRCs or some aspect of them were eligible for inclusion if they fulfilled at least one of the following criteria:   1. an external or internal evaluation of the CLAHRC(s) or CLAHRC process, 2. an exploration of the CLAHRC(s) as a novel organisational form and 3. development of theory using the CLAHRC(s) as a research setting, i.e. including empirical data. |
| Description of the public health workforce | Collaborations for Leadership in Applied Health Research and Care (CLAHRCs): collaborative partnerships between universities and surrounding National Health Service organisations, focused on improving patient outcomes through the conduct and application of applied health research. |
| Miscellaneous | 1. Conflict of interest: PMW is Deputy Editor in Chief of Implementation Science. All decisions relating to this manuscript were made by another senior editor. All authors are in receipt of funding from the NIHR CLAHRC Greater Manchester, one of 13 CLAHRCs funded in England.  2. Sponsorship/ funding : All authors are in receipt of funding from the NIHR CLAHRC Greater Manchester. The views expressed in this article are those of the authors and not necessarily those of the NHS, NIHR or the Department of Health. |
| Reference | Kislov R, Wilson PM, Knowles S, Boaden R. Learning from the emergence of NIHR Collaborations for Leadership in Applied Health Research and Care (CLAHRCs): a systematic review of evaluations. Implement Sci. 2018;13(1):111. |

1. Enabling relational leadership in primary healthcare settings: lessons from the DIALHS collaboration. July 8^th^, 2018.

| Contact detail | [Susan Cleary](javascript:;): [susan.cleary@uct.ac.za](mailto:susan.cleary@uct.ac.za) |
| --- | --- |
| Author  information | Health Economics Unit, School of Public Health and Family Medicine, University of Cape Town, Cape Town, South Africa. |
| Objective | We describe the overarching LD[leadership development] design that emerged through our collaboration, explain the governance context in which it was implemented and consider whether and how our approach to LD has, so far, enabled relational leadership. |
|  |  |
| Methods | The research approach was flexible and qualitative. Our data for this article therefore includes observational and interview notes, transcripts from reflective discussions, and written reports from the organizational psychologist. the first author read through the observational, interview and reflective discussion data as well as the documents, reports and publications from the overall DIALHS project, and extracted all data elements that spoke about management, leadership, the governance context of the sub-district and the LD interventions. Thereafter, the first author used thematic analysis to interpret the data using themes generated both from relevant bodies of literature and from the data themselves, and the initial analysis was then discussed within the author team.  In-depth interviews ( 12 interviews), reflective discussion( 7 reflective discussions, observations of LD process ( 15 group coaching processes and 1 relational leadership skills workshops observed, Report of FM [facility managers ] coaching*.* Report from external evaluator ( 1 report) and 13 interviews. |
| Description of the public health workforce | Researchers from two universities have worked with managerial colleagues from both authorities in a long-term collaborative project (DIALHS). The LD/research team that was involved in the leadership engagements included an organizational psychologist and a number of health policy and systems researchers |
| Miscellaneous | 1. Conflict of interest: *Conflict of interest statement*. None declared.  2. Sponsorship/ funding : The project is also funded by the Atlantic Philanthropies (Grant no. 18269). |
| Reference | Cleary S, Toit AD, Scott V, Gilson L. Enabling relational leadership in primary healthcare settings: lessons from the DIALHS collaboration. Health Policy Plan. 2018;33(suppl_2):ii65-ii74. |

1. Strategic leadership capacity building for Sub-Saharan African health systems and public health governance: a multi-country assessment of essential competencies and optimal design for a Pan African DrPH. July 1^st^, 2018.

| Contact detail | [Irene Akua Agyepong](https://www-ncbi-nlm-nih-gov.sheffield.idm.oclc.org/pubmed/?term=Agyepong%20IA%5BAuthor%5D&cauthor=true&cauthor_uid=30053033): [moc.liamtoh@gnopeygai](mailto:dev@null) |
| --- | --- |
| Author information. | Ghana Health Service, Division of Research and Development, Dodowa Health Research Center, Dodowa, Greater Accra, Ghana. |
| Objective | As a first step towards programme development, we undertook this assessment with two related objectives. The first was to explore more widely and empirically perceptions of the need for a pan-African DrPH, with a focus on strategic leadership. The second was to generate evidence to inform the customization of programme design to context, in terms of competencies, content and mode of delivery. |
| Methods | A mixed methods cross sectional multi-country study was conducted in Ghana, South Africa, and Uganda. Data collection involved a non-exhaustive desk review, 34 key informant (KI) interviews with past and present health sector leaders and a questionnaire with closed and open-ended items administered to 271 potential DrPH trainees.  *Document review:* Documents reviewed included peer-review articles, books and grey literature[ not mentioned the number*]. b) KI interviews* Twelve KI interviews were conducted in Ghana, four in South Africa and eighteen in Uganda. *C) Semi-structured questionnaire:* with closed and open-ended items administered to 271 potential DrPH trainees…In South Africa, a total of 111 responses were received from 400 emails inviting participation in the survey. D) *Stakeholder validation workshop:* In Ghana, a stakeholder validation workshop was held in April 2015 with 16 participants. |
| Description of the public health workforce | The Doctor of Public Health (DrPH) is a professional, interdisciplinary terminal degree focused on strategic leadership capacity building. |
| Miscellaneous | 1. Conflict of interest: *Conflict of interest statement*. None declared.  2. Sponsorship/ funding : This work was supported by Rockefeller Foundation Grant 2013 THS 307 in support of the development of a Pan African doctor of Public Health programme with a focus on strategic leadership for health systems and public health development and resilience to support health outcome improvement in Sub-Saharan Africa. |
| References | Agyepong IA, Lehmann U, Rutembemberwa E, Babich SM, Frimpong E, Kwamie A, et al. Strategic leadership capacity building for Sub-Saharan African health systems and public health governance: a multi-country assessment of essential competencies and optimal design for a Pan African DrPH. Health Policy Plan. 2018;33(suppl_2):ii35-ii49. |

1. Is blended learning and problem-based learning course design suited to develop future public health leaders? An explorative European study. June 1^st^, 2018.

| Contact detail | [Karen D. Könings](https://www-ncbi-nlm-nih-gov.sheffield.idm.oclc.org/pubmed/?term=K%26%23x000f6%3Bnings%20KD%5BAuthor%5D&cauthor=true&cauthor_uid=29881645) : [Kd.Konings@maastrichtuniversity.nl](mailto:Kd.Konings@maastrichtuniversity.nl) |
| --- | --- |
| Author  Information | Department of Educational Development and Research and Graduate School of Health Professions Education, Faculty of Health, Medicine and Life Sciences, Maastricht University, |
| Objective | Our aim was to explore learners’ perceptions of the effectiveness and appeal of a public health leadership course using problem-based, blended learning methods that used virtual learning environment technologies. |
| Methods | In this cross-sectional evaluative study, the Self-Assessment Instrument of Competencies for Public Health Leaders was administered before and after an online, blended-learning, problem-based (PBL) leadership course. An evaluation questionnaire was also used to measure perceptions of blended learning, problem-based learning, and tutor functioning.  The Self-Assessment Instrument of Competencies for Public Health Leaders (SAIC-PHL). This consisted of 52 items describing competencies essential for public health leaders. These competency descriptions are developed based on a literature review and refined and validated in a consensus development panel and two rounds of a Delphi survey. For each competency, learners had to assess how well they thought they were doing on a 5-point scale ranging from “acting as a novice” to “acting as an expert.”  All data were collected online. SPSS version 19 was used to analyze the data. Cronbach’s alpha was calculated for the different scales from the Evaluation Questionnaire and the SAIC-PHL to check whether it was acceptable to use scale scores (alpha from .70 considered as acceptable, from .80 as good).  Nineteen participants (4 males, 15 females) completed the course: eight from Maastricht University (The Netherlands), five from Kaunas University (Lithuania), and six from the Medical University of Graz (Austria). |
| Description of the public health workforce | Teachers were from the UK, Austria, Lithuania, and the Netherlands and represented various academic fields: public health, psychology, nursing, political science, education, and social science. All teachers underwent PBL training and blended learning training prior to the delivery of the course. They also served as tutors in the online tutorial groups. |
| Miscellaneous | 1. Conflict of interest: The authors declare that they have no competing interests.  2. Sponsorship/ funding : ERASMUS Multilateral Curriculum Development project: Leaders for European Public Health (LEPHIE). Project no. 510176-LLP-1-2010-1-NL-ERASMUS-ECDCE—supported by the Lifelong Learning Program of the European Commission. Funding was received for developing the course. The funding body did not have a role in design of the study and collection, analysis, and interpretation of data, nor in writing the manuscript. |
| References | Könings KD, de Jong N, Lohrmann C, Sumskas L, Smith T, O'Connor SJ, et al. Is blended learning and problem-based learning course design suited to develop future public health leaders? An explorative European study. Public Health Rev. 2018;39:13 |

1. Mapping Foreign Affairs and Global Public Health Competencies: Towards a Competency Model for Global Health Diplomacy. October 16^th^, 2016.

| Contact detail | Matthew Brown: |
| --- | --- |
| Author  information | University of California, San Diego, School of Medicine, Division of Global Public Health |
| Objective | This research identifies and maps core competencies that can be used to address this gap in the training of professionals in the fields of foreign affairs and global public health. |
| Methods | We conducted focused internet searches to identify two core competency models in foreign affairs and six competency models in global public health. First, we identified published competency models with definitions from global public health and foreign affairs training organizations by conducting web searches and literature reviews of institutions, government agencies, academic programs, and the peer-reviewed literature. Second, we created aggregate models for both foreign affairs and global public health to facilitate comparisons between the two disciplines. |
| Description of the public health workforce | GHD[global health diplomacy] is broadly defined as political activities that meet the dual goals of improving public health and strengthening relations among nations. However, linking the fields of global public health and diplomacy is a relatively recent concept, emerging over the last two decades. The concept[GHD] is relevant to global public health professionals as well as to members of the diplomatic corps.  We found no published inventories or comprehensive mapping exercises to describe competencies in the emerging field of GHD. However, we identified two core competency models that met the criteria established from our search methodology in the field of foreign affairs from a single institution, and six core competency models from the field of global public health from four different institutions |
| Miscellaneous | 1. Conflict of interest: Not provided  2. Sponsorship/ funding : Not provided |
| References | Brown M, Bergmann J, Mackey T, Eichbaum Q, McDougal L, Novotny T. Mapping foreign affairs and global public health competencies: Towards a competency model for global health diplomacy. Global Health Governance. 2016;10(2),3–49. <http://blogs.shu.edu/ghg/files/2016/10/Fall-2016-Issue-1.pdf#page=3> .Accessed 31 May 2007 |

1. Leadership for Knowledge Translation: The Case of CLAHRCs. April 22^nd^, 2015

| Contact detail | Dimitrios Spyridonidis: [Dimitrios.spyridonidis@henley.ac.uk](mailto:Dimitrios.spyridonidis@henley.ac.uk) |
| --- | --- |
| Authors information | University of Reading, United Kingdom,Imperial College Business School, London, United Kingdom and Surrey Business School, University of Surrey, Surrey, United Kingdom |
| Objective | To understand how new organizational forms for KT[ Knowledge translation] bridge the gap between research and practice, paying particular attention to the relationship between the organization and practices of KT and leadership. |
| Description of the public health workforce | This research uses multiple research methods—qualitative interviews, ethnography, and document analysis.  1. Qualitive interviews: We used semi-structured interviews with both the CORE group and PROJECT leaders. All members of the CORE group and PROJECT leaders were interviewed annually across the 5-year life span of the research. Transcripts of each interview were anonymized, and a code number was assigned to each for identification purposes. Our interest lay in exploring the motivations of different actors to get involved with the CLAHRC program, how they made sense of it, what they were seeking to achieve, how and why, and to what extent they thought that they were able to achieve their aims. The focus of interviews was broad but encompassed questions about lead actors’ backgrounds, disposition toward KT, and vision for CLAHRCs during bid development.  2. Ethnography: Finally, we observed how the CLAHRC operated in practice—as distinct from how it stated it operated in practice—in the Partnership Forum and CLD events. Field notes were written during or shortly after the periods of observation and were analyzed on return to the office. Periods of observation lasted for 2 to 8 hours at any time (total of 226 hours of non-participant observations) and included discussion with those in the field (i.e., informal interviews) to clarify aspects of KT practices. In addition to formal meetings, we observed informal conversations during breaks. During observations of formal and informal conversations, we paid attention to the nature and content of messages about KT that the CORE group espoused, and PROJECT leaders came into contact with, the content of their conversations with one another, and the nature of their interactions.  *3.* Document analysis: data collection and analysis were informed by our initial theoretical framework with both inductive and deductive approaches used rather than a pure grounded theory approach.  We used inter-coder triangulation, that is, coding of the same content by more than one coder so as to check whether the same codes get produced to assess the reliability of the coding. Inter-coder triangulation was carried out on approximately one third of all interview transcripts (*n* = 70) taken across a range of samples and phases, with coding done by two and sometimes three different researchers. |
| Description of the public health workforce | Collaborations for Leadership in Applied Health Research and Care (CLAHRCs), established in 2007 with funding from the National Institute for Health Research (NIHR). These were designed to be a new organizational form through which higher education institutions and surrounding NHS organizations collaborate to translate evidence of cost and clinical effectiveness into everyday health care.  The NIHR North West London CLAHRC describes itself as a “partnership between world class academic and clinician institutions in North West London working to build system-level translational capacity.” This involves bringing together 25 local NHS organizations (mainly primary, secondary, and associated health care providers) and local universities to form a knowledge-based network to radically transform the way clinically innovative interventions are introduced and sustained.  CLAHRC leadership consisted of two delineated groups. The first group called themselves the CORE group—this group was initially more concerned with top-down leadership—in that these were staff responsible for setting CLAHRC’s strategic vision. The CORE group consisted primarily of academics with a reputation in health services research, innovation, and policy. The CORE group was responsible for putting in place new structures, people, and clinical initiatives to encourage the translation of research findings into improved practice and thereby improve patient outcomes. The CORE group also worked with academics (organizational theorists, social scientists, health economists, and statisticians) locally, more widely in the United Kingdom, and internationally to develop and introduce quality and performance improvement methods into the local health care partners.  The second group of leaders was PROJECT leaders… All these projects involved collaboration between patients, academics, NHS frontline staff, and NHS managers…Informants were a multi-stakeholder theoretical sample, including senior and middle managers from the CORE group and individual PROJECT leaders, including project managers, doctors, nurses, and other allied health care professionals from 25 NHS organizations across primary and secondary care. Participants had a diverse understanding of knowledge and evidence-based practice. |
| Miscellaneous | 1. Conflict of interest: The authors declared no potential conflicts of interest with respect to the research, authorship, and/or publication of this article.  2. Sponsorship/ funding : The authors disclosed receipt of the following financial support for the research, authorship, and/or publication of this article: The research for this article was funded by the NIHR North West London CLAHRC. |
| References | Spyridonidis D, Hendy J, Barlow J. Leadership for Knowledge Translation: The Case of CLAHRCs. Qual Health Res. 2015;25(11):1492-505. |

1. Moving the Needle: A Retrospective Pre- and Post-analysis of Improving Perceived Abilities Across 20 Leadership Skills. August 1^st^, 2014.

| Contact detail | Cheryl C. Noble: cnoble@email.unc.edu <cnoble@email.unc.edu> |
| --- | --- |
| Author  information | Gillings School of Global Public Health University of North Carolina at Chapel Hill Chapel Hill USA |
| Objective | To assess the influence of intensive focused leadership training on self-evaluation of leadership skills among Maternal and Child Health (MCH) professionals enrolled in the Maternal and Child Health Public Health Leadership Institute (MCH PHLI). |
| Methods | Senior-level MCH leaders (n = 54) participated in the first two cohorts of the MCH PHLI, a senior-level training program funded through the Maternal and Child Health Bureau. Participants self-identified their skill level across 20 leadership skills that were the focus of the training program. These skills were derived from the MCH Leadership Competencies, 3.0 and literature reviews, and then divided into two domains: Core leadership skills and Organizational/Institutional leadership skills. |
| Description of the public health workforce | Maternal and Child Health (MCH) professionals enrolled in the Maternal and Child Health Public Health Leadership Institute (MCH PHLI).  Participants in this study were Fellows in the first two cohorts of the MCH PHLI program. Of the 55 Fellows in the first two MCH PHLI cohorts, 98 % (n = 54) successfully completed the requirements of the program and thus were eligible to participate in this study. Fellows represent a highly inter-professional group, with earned academic degrees in public health (30.9 % of the group), social work (9.1 %), law (1.8 %), medicine (9.1 %), nursing (12.7 %), education (2 %), nutrition (1.8 %), psychology (1.8 %) as well as a variety of other fields (7 %). |
| Miscellaneous | 1. Conflict of interest: Mr. Ruben Fernandez, JD, serves as a faculty in the MCH PHLI program and is related to the project Principal Investigator. The UNC conflict of interest management plan requires that all papers and presentations offer this acknowledgement, which can be included with the publication or not at the discretion of the editor.  2. Sponsorship/ funding : Not mentioned. |
| Reference | Fernandez CS, Noble CC, Jensen E, Steffen D. Moving the needle: a retrospective pre- and post-analysis of improving perceived abilities across 20 leadership skills. Matern Child Health J. 2015;19(2):343-52. |

1. Institutional capacity for health systems research in East and Central African schools of public health: knowledge translation and effective communication. June 2^nd^, 2014

| Contact detail | Richard Ayah: [richardayah@gmail.com](mailto:richardayah@gmail.com) |
| --- | --- |
| Author  information | School of Public Health, College of Health Sciences, University of Nairobi |
| Objective | This paper is based on an HSR [health systems research] capacity self-assessment that was conducted by the seven SPHs [schools of public health], with the objectives of ascertaining existing capacities for HSR; building consensus around HSR capacity development strategies for each SPH; and making an initial and rapid assessment of HSR priorities in the different countries involved in the HEALTH Alliance. |
| Methods | In 2011, each member of the Africa Hub undertook an institutional HSR capacity assessment using a context-adapted and modified self-assessment tool. KT capacity was measured by several indicators including the presence of a KT strategy, an organizational structure to support KT activities, KT skills, and institutional links with stakeholders and media. Respondents rated their opinions on the various indicators using a 5-point Likert scale. The assessment had three parts: a self-assessment, a review of internal documents in order to generate a profile of HSR in the institution, and key informant interviews of internal and external stakeholders. |
| Description of the public health workforce | The Higher Education Alliance for Leadership Through Health (HEALTH)…consortium of seven schools of public health (SPHs) in East and Central Africa: Jimma University College of Public Health and Medical Science (CPHMS, Ethiopia), Kinshasa School of Public Health (KSPH, Democratic Republic of the Congo (DRC)), Makerere School of Public Health (MakSPH, Uganda), Moi University School of Public Health (MUSOPH, Kenya), Muhimbili School of Public Health and Social Sciences (MUSPHSS, Tanzania), National University of Rwanda School of Public Health (NURSPH, Rwanda), and University of Nairobi School of Public Health (SPHUoN, Kenya).  The focal person identified key persons including the deans, deputy deans, heads of departments, and staff within the institution. Inclusion criteria were those who taught, did research, or had a stake in health system Further, to have a common understanding of HSR[health systems research]. |
| Miscellaneous | 1. Conflict of interest: The authors declare that they have no competing interests.  2. Sponsorship/ funding: This document is an output from a project funded by DFID [UK Department for International Development] for the benefit of developing countries. However, the views expressed in this document are solely the responsibility of the authors, and not necessarily those of DFID. |
| Reference | Ayah R, Jessani N, Mafuta EM. Institutional capacity for health systems research in East and Central African schools of public health: knowledge translation and effective communication. Health Res Policy Syst. 2014;12:20. |

1. In search for a public health leadership competency framework to support leadership curriculum–a consensus study, 11 October 2013

| Contact detail | [Katarzyna Czabanowska](javascript:;): kasia.czabanowska@maastrichtuniversity.nl. |
| --- | --- |
| Author  information | 1. Department of International Health, CAPHRI, Faculty of Health, Medicine and Life Sciences, Maastricht University, Maastricht, The Netherlands |
| Objective | The aim of this study was to develop a public health leadership competency framework to support the development of competency-based European public health leadership curriculum. |
| Methods | The study was carried out in three phases: a literature review, consensus development panel and Delphi survey.  *1.* Literature review carried out by two reviewers focusing on literature, which primarily addressed public health leadership in Europe, published in English between 2000 and 2011. PubMed, Cochrane Library, EMBASE and Google Scholar were accessed together with grey literature from a variety of public health and leadership institutions. The European Journal of Public Health was singled out for closer review to develop a broad understanding of leadership roles and competencies for public health professionals in continuous education programmes. Combinations of the following terms were used: a) Public health leadership competencies, b) European public health leadership competencies, c) Public health professionals’ competencies, d) European public health professional competencies, e) Public health leadership, and f) Public health leadership and continuing education.  2. Panel of experts consisting of seven public health and seven leadership academics from four European Universities. Two online consensus development panels were held to evaluate and make changes to the initial draft competency framework. Consensus development panels are a qualitative method for obtaining agreement in areas of uncertainty or where there is a lack of definitive information.  3) Delphi Survey: we chose to have two rounds of Delphi surveys in an effort to finalize and obtain a high level of consensus on the public health leadership competency framework. The first-round questionnaire consisted of 73 items describing competencies. Participants were asked to respond ‘yes/no/with changes’ on the inclusion and grouping of each competency. Space was also provided to add other competencies that respondents considered suitable for public health leadership. Items with less than 80% consensus were edited to bring them closer in line with the observations of the respondents. The second Delphi survey contained only three items and sought a simple ‘yes/no’ response regarding the edits made in the previous round. This was followed by a yes/no response regarding approval of the framework as a whole. |
| Description of the public health workforce | Panel of experts consisting of seven public health and seven leadership academics from four European Universities, and members of the ASPHER Working Group on Innovation and Good Practice in Public Health Education (WGIGP). WGIGP consists of senior public health professionals interested in education and educational leadership. |
| Miscellaneous | 1. Conflict of interest: *Conflicts of interest*: None declared.  2. Sponsorship/ funding : This study was supported by the European Commission Lifelong Learning Programme in the framework of ERASMUS Multilateral Curriculum Development project: LEPHIE. Project n° 510176-LLP-1-2010-1-NL-ERASMUS-ECDCE. This publication reflects the views only of the authors, and the Commission cannot be held responsible for any use that may be made of the information contained herein. |
| References | Czabanowska K, Smith T, Könings KD, Sumskas L, Otok R, Bjegovic-Mikanovic V, et al. In search for a public health leadership competency framework to support leadership curriculum-a consensus study. Eur J Public Health. 2014;24(5):850-6. |

1. Evaluation of leadership Competencies of executives in Lithuanian public Health Institutions. November 5^th^ ,2012.

| Contact detail | Mindaugas STANKŪNAS: [mindstan@gmail.com](mailto:mindstan@gmail.com) |
| --- | --- |
| Author  information | 1. School of Public Health, Griffith University, Gold Coast Campus, Queensland, Australia. |
| Objective | the aim of this study was to explore the self-assessed level of leadership competencies of executives in Lithuanian public health institutions. |
| Methods | A cross-sectional survey of executives of Lithuanian public health institutions in 2010. Respondents were asked about their competencies in leadership, teamwork, communication, and conflict management. The evaluation was carried out by analyzing the answers provided in the survey, which used a 5-point rating scale. In addition, the Belbin Team-Role Self-Perception Inventory and the Thomas-Kilmann Conflict Mode Instrument were used. Data were coded and analyzed with the SPSS (version 13.0) using descriptive statistical analysis methods. |
| Description of the public health workforce | Questionnaires were distributed to all executives (chief executive officers, their deputies, and heads of territorial branches) of all Lithuanian public health institutions (public health centers and public health bureaus). |
| Miscellaneous | 1. Conflict of interest: The authors state no conflict of interest.  2. Sponsorship/ funding : The paper does not mention sponsorship or funding. |
| References | Stankūnas M, Sauliūnė S, Smith T, Avery M, Šumskas L, Czabanowska K. Evaluation of leadership competencies of executives in Lithuanian public health institutions. Medicina (Kaunas). 2012;48(11):581-7. |

1. Competency-Based Impact of a Statewide Public Health Leadership Training Program. February 15, 2010.

| Contact detail | Suzanne R. Hawley :[shawley@kumc.edu](mailto:shawley@kumc.edu) |
| --- | --- |
| Author  information | Department of Preventive Medicine and Public Health at the University of Kansas School of Medicine-Wichita, Wichita, Kansas. |
| Objective | The present study describes the impact of the KPHLI [Kansas Public Health Leadership Institute] training process on the self-reported public health core competencies and public health leadership competencies of scholars, with further analysis of competency changes for specific workforce demographics. |
| Methods | Identical assessments, including the two competency assessments, were administered online to participants pre and post KPHLI training. Data were statistically analyzed in SPSS/PC 14.0 using Wilcoxon signed ranks tests to note areas of significant improvement following training. After analysis of the overall pool, data were stratified according to years of public health experience, level of education, and urban or rural status, and correlations were calculated using Spearman’s rho tests in SPSS/PC 14.0. |
| Description of the public health workforce | Kansas has implemented practitioner-oriented training through the Kansas Public Health Leadership Institute (KPHLI).The first four cohorts of KPHLI [Kansas Public Health Leadership Institute] scholars (N = 109) were drawn from public health and allied health agencies in 32 counties across the state of Kansas, 20 of which are rural or frontier. |
| Miscellaneous | 1. Conflict of interest: Not provided.  2. Sponsorship/ funding: Not provided. |
| Reference | Hawley SR, St Romain T, Orr SA, Molgaard CA, Kabler BS. Competency-based impact of a statewide public health leadership training program. Health Promot Pract. 2011;12(2):202-8. |

**Supplementary material 4. Domains for the competencies in the framework.**

| ***Domain.*** | ***Definition or description of the domains.*** | ***Reference.*** |
| --- | --- | --- |
| Change management | ¨Creating and managing (organisational/culture) change, shifting conditions from a baseline to goal state. ¨ | Bayley J, Phipps D, Batac M, Stevens E. Development of a framework for knowledge mobilisation and impact competencies. Evidence & Policy*.*2018; *14*(4): 725-738.doi: 10.1332/174426417X14945838375124 |
| Communication | ¨Communicating with a range of stakeholders, both internally and externally, individually and in teams. ¨ | Bayley J, Phipps D, Batac M, Stevens E. Development of a framework for knowledge mobilisation and impact competencies. Evidence & Policy*.*2018; *14*(4): 725-738.doi: 10.1332/174426417X14945838375124 |
| Training and capacity building | ¨Supporting the development of KT skills and understanding, improving individual and organisational competency. ¨ | Bayley J, Phipps D, Batac M, Stevens E. Development of a framework for knowledge mobilisation and impact competencies. Evidence & Policy*.*2018; *14*(4): 725-738.doi: 10.1332/174426417X14945838375124 |
| Knowledge management | ¨Offering users valid information tailored to their settings and needs. ¨ | Dagenais C, Laurendeau MC, Briand-Lamarche M. Knowledge brokering in public health: A critical analysis of the results of a qualitative evaluation. Eval Program Plann. 2015;53:10-7. |
| Engagement diverse others in public health work | ¨Engaging others requires searching broadly for partners, understanding their view world and practical circumstances, outlining potential responses, and identifying the right partners for a particular activity. ¨ | Begun, J and Peak, S. *Leading public health: A competency framework [electronic resource] (1^st^ edition)*. New York: Springer Publishing Company;2014. eBook ISBN 9780826199072 |

**Supplementary material 5. Competencies that were considered over the Delphi study (Not shown competencies that reached consensus).**

| Number | Competency |
| --- | --- |
| 1 | Participate in public health policy initiative at the local, national and/or international levels. |
| 2 | Advocate in public health initiatives at the local, national and/or international levels using their influence and advocacy platforms. |
| 3 | Evaluate and determine appropriate actions regarding critical political issues. |
| 4 | Understand and apply a range of knowledge translation approaches and efforts to promote cultural change and freedom to experiment, learn and adapt. |
| 5 | Build leadership capacity for knowledge translation, based on developing competencies in epidemiology, research methods, communication, policy, critical appraisal and health economics. |
| 6 | Guide organisational decision-making processes and planning informed by evidence. |
| 7 | Develop and manage specific knowledge translation tools to facilitate shared decision making for health among stakeholders. |
| 8 | Develop and manage a statistical process to monitor and control knowledge translation mechanisms. |
| 9 | Develop and manage frameworks and/or models to support continuous quality improvement of knowledge translation |
| 10 | Develop and manage knowledge translation process mapping tools to display a model, framework or cycle to conduct knowledge translation |
| 11 | Develop and manage web-based methods to disseminate evidence to stakeholders and strengthen communication between them. |
| 12 | Ensure that organisational practices are aligned with changes to pursue social justice and equity in the public health system and the larger social, political and economic environment. |
| 13 | Build, maintain and consolidate alliances, partnerships, and coalitions to improve the health of the community or population being served. |
| 14 | Make strategic decisions based on recognised values, priorities and resources. |
| 15 | Understand the nature of the public health issues and consider the policy options to engage in systemic change |
| 16 | Support and recognise knowledge brokers functions in the health system, including a clear job description supported by competencies for research and evidence management. |
| 17 | Influence public health policies and resources allocation decisions within political, economic and social system context at a local, national, or international level. |

1. OPRENGREY the author has not applied date limit [↑](#footnote-ref-1)
2. Spanish was not an option for limit. [↑](#footnote-ref-2)
